# Early Detection of Secondary Bladder Urothelial Carcinoma and Disseminated Bone Metastases with Normal Prostate-Specific Antigen Level after Pelvic Salvage Radiotherapy in Prostate Cancer

**DOI:** 10.3390/life13061249

**Published:** 2023-05-25

**Authors:** Teak Jun Shin, Misun Choe, Byung Hoon Kim, Sang Jun Byun

**Affiliations:** 1 Department of Urology, Keimyung University School of Medicine, Daegu 42601, Republic of Korea; teakjunshin@dsmc.or.kr (T.J.S.); blackporori@dsmc.or.kr (B.H.K.); 2 Department of Pathology, Keimyung University School of Medicine, Daegu 42601, Republic of Korea; msc@dsmc.or.kr; 3 Department of Radiation Oncology, Keimyung University School of Medicine, Daegu 42601, Republic of Korea

**Keywords:** prostate cancer, pelvic radiotherapy, secondary bladder cancer, bone metastases, normal prostate-specific antigen

## Abstract

This report describes the case of a 65-year-old man who presented with gross hematuria and a history of pelvic salvage radiotherapy for prostate cancer. Cystoscopy and transurethral resection of the bladder revealed urothelial carcinoma. Subsequently, disseminated bone metastases were detected with normal prostate-specific antigen (PSA) levels, and palliative radiotherapy and systemic chemotherapy were administered. Because gross hematuria can appear in both acute/chronic cystitis and bladder cancer in patients who have undergone pelvic radiotherapy for prostate cancer, close follow-up along with a detailed evaluation is needed. In addition, because prostate cancer disease progression with normal PSA levels may be associated with specific pathological findings, a detailed evaluation of symptoms and a careful review of pathologic reports are important.

## 1. Introduction

External beam radiation therapy (EBRT) is applied and implemented in various ways for definitive or palliative purposes for most solid tumors [1]. The principle of EBRT is to break down tumor DNA through irradiation of the body; however, radiation inevitably affects nearby organs or tissues [2]. Various radiation-induced complications can affect the patient’s quality of life during and after radiotherapy. Recently developed intensity-modulated radiation therapy (IMRT) can minimize the dose to surrounding tissues while irradiating the treatment area with the highest dose possible, and it is thus becoming commonly used [3]. Along with the IMRT technique, especially in EBRT for prostate cancer, which has a low alpha/beta ratio, dose escalation and hypofractionation have become possible while minimizing radiation exposure to the surrounding organs [4].

Cystitis is the most common acute complication after pelvic radiotherapy for prostate cancer, and secondary malignancy is a serious chronic complication [5]. However, gross hematuria may also be observed in both cystitis and bladder cancer. Therefore, in patients who have undergone pelvic radiotherapy for prostate cancer, close follow-up is needed, and time-appropriate differential diagnosis is important.

Prostate-specific antigen (PSA) is mainly expressed in the prostate, and serum PSA levels usually reflect the presence of prostate tissue in the body [6]. Therefore, PSA is used to suggest disease activity or status in patients with prostate cancer and is an invaluable tool not only for initial diagnosis but also for monitoring after treatments [7]. A short PSA doubling time and rapid increase in serum PSA levels are associated with early disease progression and poor survival rates, whereas undetectable to normal range PSA levels are related to better disease-free survival rates [6]. Therefore, disease progression in prostate cancer with undetectable or normal PSA levels is very rare and has only been reported in selected cases [6,8,9].

Here, we report a case of early detected bladder urothelial carcinoma and disseminated bone metastases with normal PSA levels during the follow-up period after EBRT for biochemical recurrence, which occurred after prostatectomy; this is the first report of these two sequential events in a single patient.

## 2. Detailed Case Description

A 65-year-old man underwent prostatectomy for prostate cancer at another local hospital and continued regular follow-ups for serum PSA levels. The patient had no medical comorbidities and was an ex-smoker (smoked two packs per day for 20 years and stopped 23 years ago). Prostate magnetic resonance imaging (MRI) before prostatectomy showed definite extra-prostatic extension, seminal vesicle invasion, and no significant lymph node enlargement (cT3bN0). After prostatectomy, a poorly differentiated adenocarcinoma was diagnosed, and seminal vesicle invasion was observed (pT3b) with a Gleason score of 9 (5 + 4). The tumor occupied 90% of the prostate volume; the resection margin was positive. It also revealed lymphovascular invasion, perineural invasion, and a cribriform pattern of tumor cells. The initial PSA level before prostatectomy was 28.3 ng/mL and the nadir, at 1 month post-surgery, was 0.173 ng/mL. During the subsequent follow-up period, increased PSA levels of 0.437 and 0.635 ng/mL were observed at 3 and 5 months post-surgery, respectively. Prostate MRI and whole-body bone scanning (WBBS) were performed to rule out local recurrence or distant bone metastasis, which showed no specific findings; the patient was considered to demonstrate biochemical recurrence (BCR) 5 months following prostatectomy [10]. Androgen deprivation therapy (ADT), including gonadotropin-releasing hormone agonists and androgen antagonists, was initiated, and the patient was referred to our institution for salvage radiotherapy.

**Figure 1:**
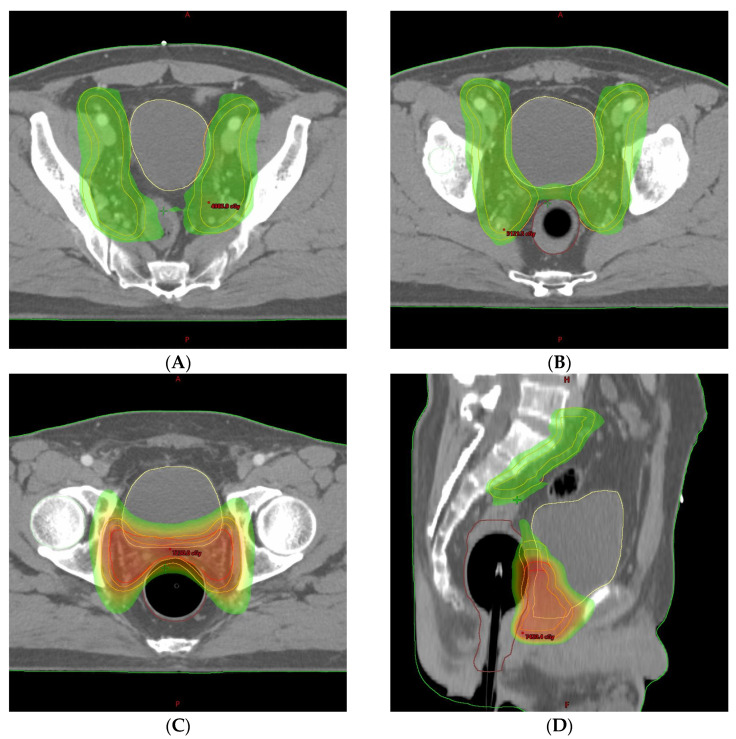
Pelvic radiotherapy dose distribution. The planned dose distribution in (**A**–**C**) the axial and (**D**) sagittal planes are shown. Colored areas indicate a planned radiation dose of 44 Gy (yellow–green) or 66 Gy (red).

Before treatment planning computed tomography (CT) and daily radiotherapy, the bladder was prepared by water intake, followed by rectal ballooning according to our institution’s protocol. This preparation protocol aimed to limit the radiation dose to the small bowel, bladder, and rectum. The bladder volume was prepared to be 180–250 cc by drinking sufficient water 1 h before the treatment planning CT and checking the bladder volume using an ultrasound scanner. A balloon catheter for radiotherapy was inserted into the rectum; 60 cc of air was injected. For treatment planning, the clinical target volume (CTV) was delineated according to the Radiation Therapy Oncology Group (RTOG) guidelines [11]. The upper border of the pelvic CTV began from the L5–S1 interspace, and the common iliac, external, and internal iliac nodal regions were included. A 7 mm margin was adopted around the vessels to create a pelvic nodal CTV; the planning target volume was an all-direction expansion of 5 mm from the CTV, except for the posterior direction of 3 mm. The patient underwent salvage radiotherapy with an IMRT plan of a dose of 66 Gy in 30 fractions for 40 days, which was split by 44 Gy in 20 fractions to the pelvic nodal area and prostate bed, followed by 22 Gy in 10 fractions to the prostate bed only (Figure 1). No particularly serious acute gastrointestinal or genitourinary complications occurred during radiation treatment. The PSA level before radiotherapy was 0.237 ng/mL, which was decreased to <0.01 ng/mL after radiotherapy completion.

**Figure 2:**
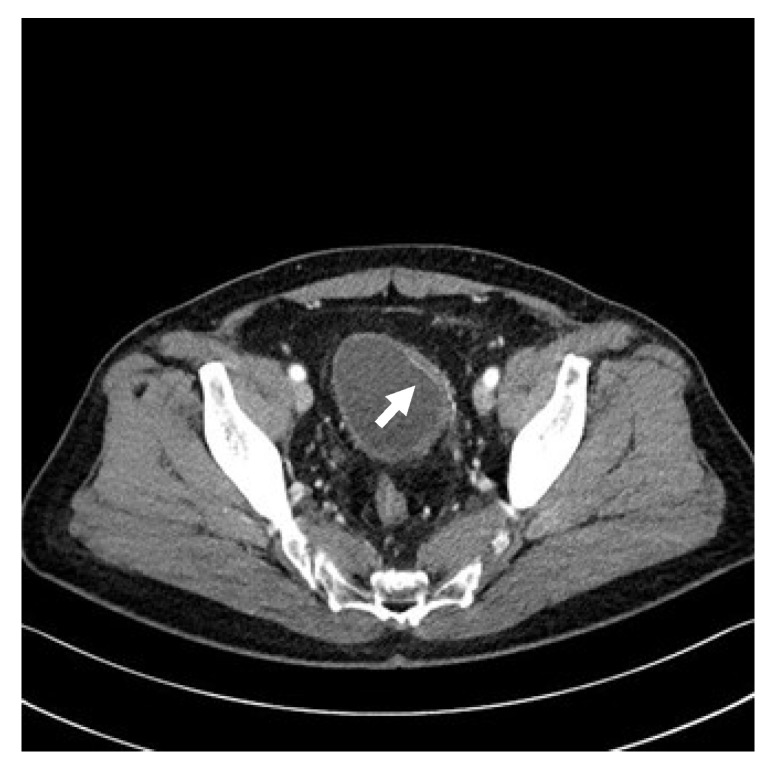
Pelvic computed tomography. Pelvic computed tomography reveals a mild bladder wall thickening with prominent mucosal enhancement (white arrow).

**Figure 3:**
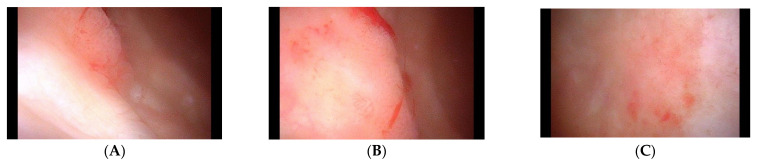
Cystoscopy of the bladder. Cystoscopy shows (**A**,**B**) a papillary lesion on the right lateral wall, and (**C**) a diffuse erythematous lesion on the posterior wall.

**Figure 4:**
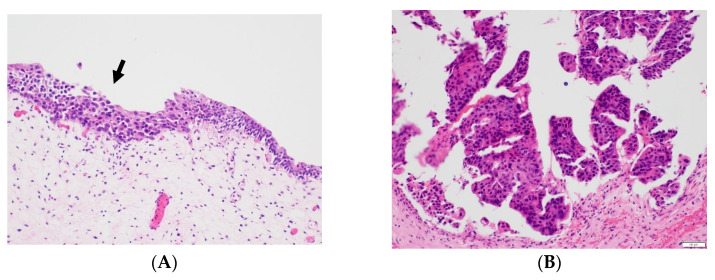
Transurethral resection of the bladder (TURB). Pathological report post-TURB shows (**A**) urothelial carcinoma in situ (black arrow) and (**B**) high-grade non-invasive papillary urothelial carcinoma [H&E stain, ×200].

The patient visited a radiation oncologist regularly for follow-up; painless gross hematuria was noticed 6 months after radiotherapy completion (15 months following prostatectomy). The hemoglobin level was 11.5 g/dL, urinalysis revealed positive erythrocytes, and the PSA level remained <0.01 ng/mL. Pelvic CT showed bladder wall thickening, and cystitis was suspected (Figure 2). Atypical cells with neoplastic characteristics were reported in urine cytology. The patient was immediately referred to our urologist for additional cystoscopy to verify the bladder lesions. On cystoscopy, no specific findings were observed in the bladder’s left lateral wall, the thick area seen on CT. However, cystoscopy showed a 1 × 1 cm flat papillary lesion in the right lateral wall and a 2 × 2 cm diffuse erythematous lesion in the posterior wall, which were not observed on the CT (Figure 3). Furthermore, the urologist performed transurethral resection of the bladder (TURB). In the pathologic report after TURB with complete resection, urothelial carcinoma in situ and high-grade non-invasive papillary urothelial carcinoma were diagnosed in the two lesions on cystoscopy (Figure 4). The patient also underwent six cycles of Bacillus Calmette–Guérin (BCG) instillation for 6 weeks; follow-up was continued.

**Figure 5:**
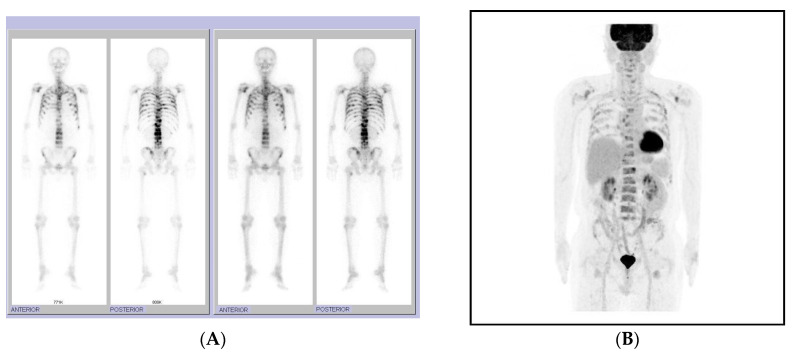
Whole-body bone scanning (WBBS) and positron emission tomography/CT (PET/CT) show systemic disseminated bone metastases. (**A**) WBBS reveals multifocal tracer uptake in the axial skeleton, ribs, sternum, scapulae, pelvic bones, and femurs, suggesting systemic bone metastasis. (**B**) An 18-FDG PET/CT reveals disseminated hypermetabolic lesions in the entire spine, ribs, scapulae, pelvic bones, sternum, and proximal humeri and femurs.

**Figure 6:**
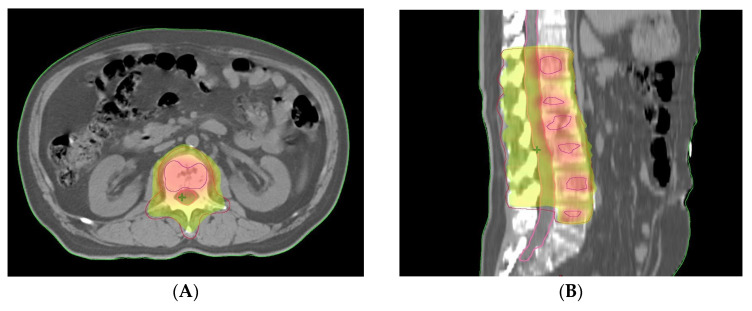
Intensity-modulated radiation therapy with the simultaneous integrated boost. Radiotherapy dose distribution in the (**A**) axial and (**B**) sagittal planes PET/CT scans are shown. Red area indicates the gross target volume: hypermetabolic lesions irradiated with a total dose of 40 Gy in 10 fractions. Yellow–green areas indicate the clinical target volume: residual vertebrae and spinous processes with a total dose of 30 Gy in 10 fractions.

Two months after BCG instillation for bladder cancer, the patient visited orthopedics for lower back pain; a spinal MRI revealed multiple spine metastases, mainly in the lumbar spine. Additional WBBS and fluorine-18 fluorodeoxyglucose positron emission tomography/computed tomography (^18^F-FDG PET/CT) showed disseminated bone metastases throughout the body 14 months after radiotherapy completion (23 months following prostatectomy) (Figure 5). At that time, the PSA and testosterone levels were 1.1 ng/mL and 0.14 ng/mL. Other tumor marker levels, including alpha-fetoprotein (2.2 ng/mL), carcinoembryonic antigen (CEA, 0.6 ng/mL), carbohydrate antigen 19-9 (CA 19-9, 2.2 U/mL), and carbohydrate antigen 15-3 (CA 15-3, 2.8 U/mL) were in normal ranges. For lower back pain, intravenous and oral analgesics were administered, and palliative radiotherapy was administered to the lumbar spine. Because of a pelvic radiotherapy history for prostate cancer, 40 Gy in 10 fractions of IMRT was planned with the simultaneous integrated boost technique to limit the dose to the spinal cord and surrounding organs (Figure 6). After palliative radiotherapy completion, lower back pain was relieved. Finally, we assessed the metastatic castration-resistant prostate cancer at the normal PSA range; therefore, we initiated chemotherapy, and the patient has currently finished 2 cycles of intravenous docetaxel. The patient is awaiting the next cycle of chemotherapy under supportive care and blood transfusion owing to pancytopenia. It has been exactly 2 years since the initial treatment, prostatectomy, and the patient has been showing poor progress and prognosis, such as BCR after 5 months and bone metastasis after 23 months.

## 3. Discussion

We described a bladder urothelial carcinoma that occurred 6 months after pelvic salvage radiotherapy, followed by systemic bone metastases at normal PSA level in a patient with prostate cancer.

Patients undergoing pelvic radiotherapy are at high bladder damage risk [12]. Bladder-related complications include increased urinary frequency, urinary urgency, dysuria in mild cases, and cystitis with persistent hematuria and secondary bladder cancer in severe cases [5]. These complications are classified as acute or chronic depending on the time they developed; furthermore, RTOG and the European Organization for Research and Treatment of Cancer criteria are used to grade the complications [13]. Complications including acute cystitis are mild in most patients, ranging as grade 1–2 based on the RTOG criteria; most patients show self-limiting symptom courses without other treatments [14]. However, the late radiation cystitis incidence is approximately 18% and can occur during 6 months–20 years [15]. Additionally, depending on symptom severity, it may require invasive procedures or lead to a life-threatening situation that decreases patients’ quality of life. Secondary bladder cancer can also cause gross hematuria as a moderate-to-severe complication in patients who received pelvic radiotherapy [16]. Therefore, particularly in patients with post-pelvic radiotherapy gross hematuria, differentiation of cystitis from secondary bladder cancer and cystoscopy is necessary.

Here, we initially performed non-invasive tests, including urine cytology and CT, for the gross hematuria observed during follow-up after pelvic radiotherapy. Next, we used cystoscopy to evaluate the wall-thickening lesion of the bladder noted on the CT scan. Therefore, early bladder cancer was diagnosed on CT-invisible lesions, and maximal TURB followed by BCG instillation treatment was completed. However, prior to cystoscopy for differentiating between hemorrhagic cystitis and secondary bladder cancer, regular urinalysis during and after radiotherapy would be helpful in detecting microhematuria associated with acute cystitis before progressing to chronic cystitis with gross hematuria.

Several retrospective studies have investigated the risk, incidence, and latency of bladder cancer secondary to pelvic radiotherapy for prostate cancer [17,18,19]. According to Moon et al., the hazard ratio (HR) of secondary bladder cancer post-radiotherapy was 1.6, and the bladder was the only organ with an increased risk of secondary cancer after brachytherapy [17]. Another study from the Cancer of the Prostate Strategic Urologic Research Endeavor database showed that the risk of secondary bladder cancer post radiotherapy was approximately two-fold higher than that post surgery [18]. Huang et al. analyzed the risk of secondary malignancy post-surgery and post-radiotherapy; the HR increased from 1.86 at 5 years to 4.94 at 10 years after completion of radiotherapy [19]. The incidence of post-radiotherapy bladder cancer in prostate cancer was 1–3%, and a previous analysis showed a two-fold increased risk of bladder cancer in patients with prostate cancer post-pelvic radiotherapy [12]. Chrouser et al. retrospectively analyzed the latency period, ranging between 6 months to 7 years and Murray et al. also reported a mean value of 4 to 5 years of latency between radiation exposure and the clinical manifestation of malignancy [20,21]. Along with the increased risk of bladder cancer, patients who have received radiotherapy are at high risk of high-grade tumors at the time of diagnosis. According to reports on patients with a history of radiotherapy for prostate cancer, 92% had a high-grade tumor compared to 77% of patients without a history of radiotherapy [22]. Moreover, the patients treated with pelvic radiotherapy were 2.7 times more likely to develop muscle-invasive bladder cancer than those treated without pelvic radiotherapy. However, not all studies have reported an association between secondary cancer risk and pelvic radiotherapy. The Mayo Clinic study reported no risk of radiation-induced secondary bladder cancer after pelvic radiotherapy [20]. The risk of secondary bladder cancer did not increase after adjuvant radiotherapy for prostate cancer, and the authors concluded that the reason for this was the dose difference between adjuvant and definitive radiotherapy.

Post-radiotherapy secondary bladder cancer has been reported for other pelvic cancers, including endometrial, rectal, and prostate cancers. Guan et al. analyzed 24,522 patients with rectal cancer treated with surgery and radiotherapy and 50,124 treated with surgery alone from the Surveillance, Epidemiology, and End Results (SEER) database [23]. Secondary bladder cancer incidence was 1.85% for radiotherapy and 1.24% for surgery alone, and the risk of secondary bladder cancer was significantly related to previous pelvic radiotherapy. However, overall and cancer-specific survival rates in patients with secondary bladder cancer after radiotherapy were comparable to those in patients who did not receive radiotherapy. According to an analysis by Wen et al. using the SEER database, secondary bladder cancer is related to radiotherapy in endometrial cancer [24]. They reported that the incidence of secondary bladder cancer was 1.76%, 1.31%, and 0.96% in patients with EBRT, brachytherapy, and others, respectively, and both the EBRT (standardized incidence ratio (SIR) = 2.24, 95% confidence interval (CI) [1.94–2.58]) and brachytherapy (SIR = 1.76, 95% CI [1.44–2.13]) groups had a higher incidence of secondary bladder cancer than did the general population. The competing risk analysis showed that brachytherapy (HR = 1.46, 95% CI [1.14–1.87]) and EBRT (HR = 1.97, 95% CI [1.64–2.36]) were independent risk factors for secondary bladder cancer.

Here, bladder cancer was diagnosed as gross hematuria 6 months after radiotherapy completion, which is a secondary bladder cancer that occurred relatively early compared to previously reported results [20,21]. Additionally to pelvic radiotherapy, we speculate that smoking history, a well-known risk factor, is another possible cause of bladder cancer. Although the patient had quit smoking 23 years prior, he was a heavy smoker (40 pack-years). In previous reports, the risk of high-grade superficial bladder cancer in patients with a ≥40 pack-year smoking history compared to non-smokers was 4.2 [25]. The risk of high-grade superficial cancer was also 1.4 times higher than in non-smokers, even if patients had quit smoking for ≥20 years.

Generally, disease progression and PSA levels are closely related in patients with prostate cancer [26]. Moreover, owing to the high negative predictive value of PSA levels, patients with undetectable post-treatment PSA levels can be considered disease-free. However, few retrospective analyses or case reports describe disease progression despite normal or undetectable PSA levels [6,8,9]. Among 394 patients who underwent prostatectomy, 2.3% presenting with undetectable PSA levels developed metastasis [8]. Leibovici et al. retrospectively analyzed 46 patients with prostate cancer who showed disease progression to metastases after the initial treatment and reported that the 46 patients were approximately 1.1% of all patients treated for prostate cancer at their institution during the same period [6]. Twenty-three, eleven, and twelve patients underwent radical prostatectomy, radiotherapy, and ADT as the initial therapy, respectively. The range of PSA levels was 0.1–2.0 ng/mL at the time of metastasis. They suggested that atypical histologic variants, particularly small cell and ductal carcinoma, Gleason scores > 7, and locally advanced tumors are characteristics related to prostate cancer progression despite undetectable PSA levels. Another study analyzed eight patients with metastatic prostate cancer with PSA levels < 10 ng/mL [9]. Immunohistochemical staining showed poorly differentiated or undifferentiated carcinoma in seven cases and moderately differentiated carcinoma in only one case. Moreover, elevated CEA levels were associated with poorly differentiated prostate adenocarcinoma. Consistently, Kageyama et al. reported that serum PSA levels are not proportional to prostate cancer progression because undifferentiated and poorly differentiated carcinomas lose the characteristics of the original prostate tissue [27].

Herein, the laboratory tests, including PSA and several tumor markers, remained within normal ranges despite systemic multiple bone metastases. Compared to previous reports regarding bone metastases with undetectable or normal PSA levels, our case showed two notable possible risk factors, including locally advanced disease and a Gleason score of 9 (highly poorly differentiated carcinoma). However, without the lower back pain presentation, the detection of bone metastases would have been further delayed. Especially in this rare case, prostate-specific membrane antigen (PSMA) PET/CT, which has been widely used recently, would also be helpful in determining the treatment progress of patients with prostate cancer at an early stage [28].

## 4. Conclusions

Hematuria is a common complication occurring post-pelvic radiotherapy for prostate cancer and may indicate acute/chronic cystitis or secondary bladder cancer. Therefore, continuous examination for hematuria during post-pelvic radiotherapy follow-up, monitored by careful examination using cystoscopy when symptoms appear, and histological examination of suspected lesions may be helpful in the early diagnosis of secondary bladder cancer.

Disease progression with undetectable or normal PSA level is a rare subset of castration-resistant prostate cancer. Close observation and imaging studies based on careful physical examination, including PSMA PET/CT, may be helpful in patients with prostate cancer characterized by locally advanced tumors or high-risk pathological features.

## Data Availability

Department of Radiation Oncology, Keimyung University Dongsan Hospital, Daegu, Republic of Korea.
